# Recent Advances in Developing Lanthanide Metal–Organic Frameworks for Ratiometric Fluorescent Sensing

**DOI:** 10.3389/fchem.2020.624592

**Published:** 2021-01-25

**Authors:** Tianying Sun, Yaobin Gao, Yangyang Du, Lei Zhou, Xian Chen

**Affiliations:** ^1^ School of Chemical Engineering and Technology/School of Marine Sciences, Sun Yat-sen University, Zhuhai, China; ^2^ College of Materials Science and Engineering, Shenzhen University, Shenzhen, China

**Keywords:** lanthanide, luminescence, dual emission, ratiometric sensing, metal–organic frameworks

## Abstract

Fluorescent probes have attracted special attention in developing optical sensor systems due to their reliable and rapid fluorescent response upon reaction with the analyte. Comparing to traditional fluorescent sensing systems that employ the intensity of only a single emission, ratiometric fluorescent sensors exhibit higher sensitivity and allow fast visual screening of analytes because of quantitatively analyzing analytes through the emission intensity ratio at two or more wavelengths. Lanthanide metal–organic frameworks (LnMOFs) are highly designable multifunctional luminescent materials as lanthanide ions, organic ligands, and guest metal ions or chromophores are all potential sources for luminescence. They thus have been widely employed as ratiometric fluorescent sensors. This mini review summarized the basic concept, optical features, construction strategies, and the ratiometric fluorescent sensing mechanisms of dual-emitting LnMOFs. The review ends with a discussion on the prospects, challenges, and new direction in designing LnMOF-based ratiometric fluorescent sensors.

## Introduction

Fluorescent probes have attracted special attention in developing optical sensor systems due to their reliable and rapid fluorescent response upon reaction with the analyte (Vendrell et al., 2012). The strategy to develop effective fluorescent probes has become one of the hottest research areas in recent years. Various kinds of fluorescence probes based on organic dye molecules, semiconductor quantum dots (QDs) have been developed (Wu et al., 2020a; Yang et al., 2020a; Pfeifer et al., 2020). However, these probes have several limitations in detecting or sensing. For example, organic dyes suffer from several drawbacks such as poor chemical stability and rapid photobleaching, making it impossible for long-term fluorescence sensing. Compared with organic dye molecules, QDs exhibit better chemical and photostability. QDs are usually composed of heavy metals and suffer from toxicity and environmental hazards, which limit their practical applications. Lanthanide-based fluorescent probes can surpass the aforementioned inherent limitations of organic dyes or quantum dots due to their excellent properties, including low toxicity and better stability. Moreover, lanthanide luminescence features rich linelike emission bands, large Stokes shift, and high resistance to photobleaching, making the lanthanide-based luminescent materials superior in sensing applications.

Traditional fluorescent sensing system that employs the intensity of only a single emission for quantitative analysis of analytes can be difficult and might yield unreliable results. A number of analyte-independent factors such as instrumental drift, the microenvironment and local concentration variance of probes, and photobleaching of the probes, all interfere with the quantification of the analyte and lower the sensing reliability. To circumvent these drawbacks, a ratiometric approach has been adopted in the design of fluorescent sensors (Chen et al., 2020; Zhao and Li, 2020). Ratiometric fluorescent sensors allow the simultaneous measurement of emission intensities at two or more wavelengths, and their emission intensity ratio is calculated and then correlated to analytes. In particular scenarios, change in emission color output could be observed upon reaction with the analyte, allowing for fast visual screening of analytes (Chen et al., 2018; Su et al., 2018; Bigdeli et al., 2019; Zhang et al., 2020). Perceiving the variation in the color brightness is much harder than visualizing the color change. Therefore, the ratiometric fluorescence sensing method is a clear winning strategy over the traditional single emission intensity-based sensing approach.

The search for novel lanthanide-based dual- or multi-emitting materials that suitable for developing a ratiometric sensor is currently an emerging field (Yin and Yin, 2020). Lanthanide metal–organic frameworks (LnMOFs) combine the lanthanide luminescent features with the MOFs’ characteristics such as high porosity, large surface-to-volume ratio, diverse structures have been proved to be a powerful candidate in sensing application (Zhang et al., 2018; Liu et al., 2019). The permanent porosity and large surface area provide the potential to effectively concentrate analytes at higher levels within MOFs (Han et al., 2012), thus improve the sensitivity. Comparing to other conventional lanthanide hybrid platforms such as lanthanide–QDs or lanthanide–dye, LnMOFs present superior emission tuning capability as lanthanide nodes, organic linkers, the ligand to metal energy transfer, all of which can be “tailor-made” or modulated (Yan, 2017; Yang et al., 2020b; Wu et al., 2020c). Moreover, the intrinsic porosity of MOFs provides a pathway to encapsulate guest materials, which may also contribute to emission. Furthermore, the host framework–guest interactions such as coordination bonds, π–π interaction, and hydrogen bonding provide excellent sensing sensitivity toward the analyte (Xu and Yan, 2017). Therefore, all these features make LnMOFs promising in developing ratiometric sensing systems.

In this mini review, we provide a general overview of the design principles of LnMOFs with multiple luminescent centers. To further discuss the details in design and the working mechanisms of these ratiometric fluorescent sensors, very recent progress in the development of LnMOFs for sensing ions, trace-water, gas and temperature, molecular decoding and biosensing will also be discussed. Our efforts here are to highlight the prospects of LnMOFs as ratiometric fluorescent sensors and briefly discuss the desired properties for future development.

### The Construction of Dual-Emitting LnMOFs

**FIGURE 1:**
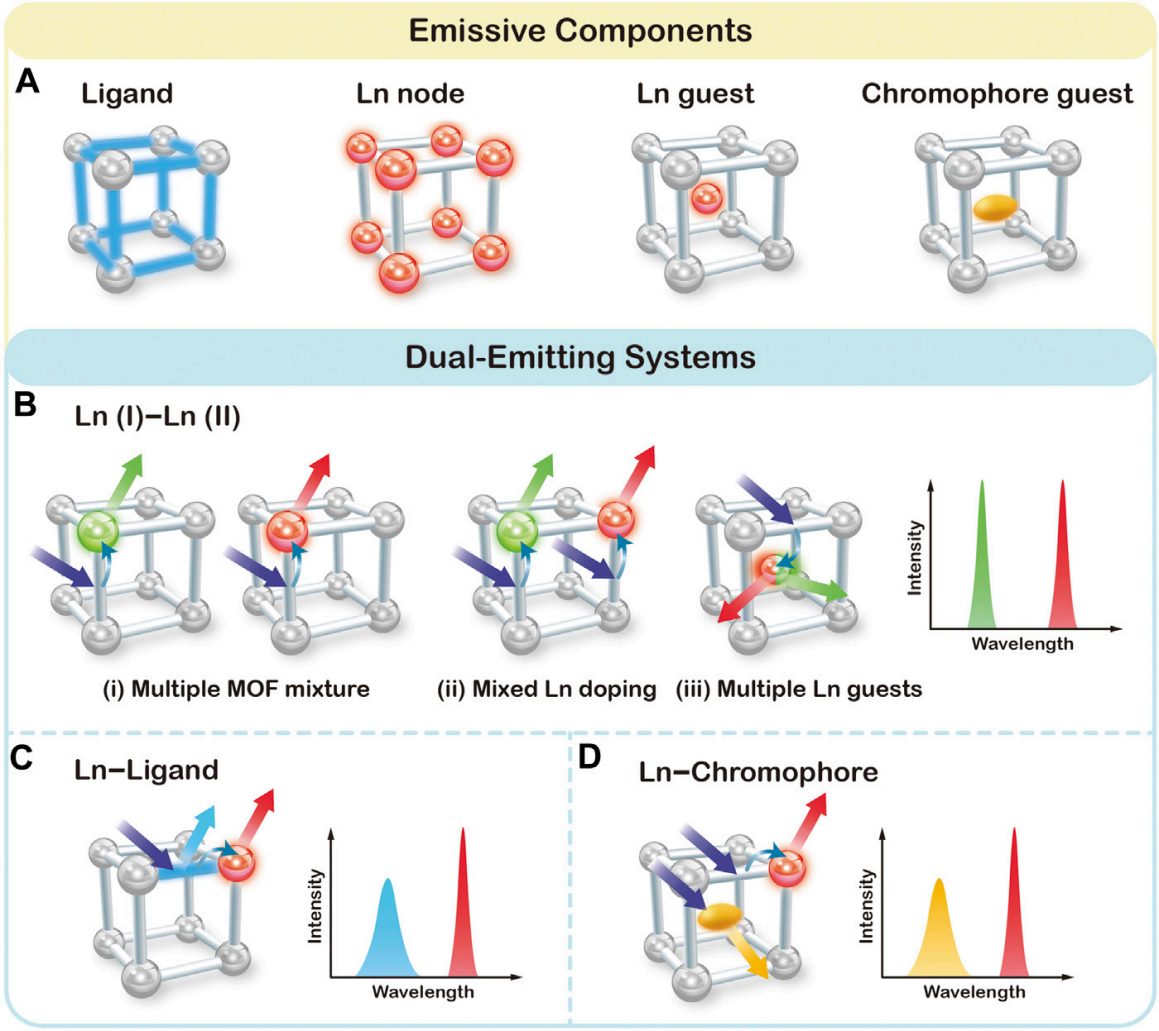
Schematic representation of four possible emissive components **(A)**, and three typical scenarios for the construction of dual-emitting ratiometric fluorescent sensors in LnMOFs **(B–D)**.

The prerequisite of ratiometric assays is to generate dual or multi emission signals by one excitation. By virtue of the inherent hybrid nature, LnMOFs can mostly generate luminescent from the following component (Figure 1A): 1) Ligand usually conjugated organic compounds can generate emission upon excitation with UV or visible light. 2) Lanthanide metal nodes can give emission through antenna effect promoted by the ligands. 3) Lanthanide ions as guest species that incorporated into MOF structures could also yield luminescence. 4) Other chromophore guests, such as dye molecules or QDs. Figures 1B–D summarizes three typical scenarios by which dual emission has been generated and utilized for the construction of ratiometric sensor, namely a Ln (I)–Ln (II) bimetallic system, a Ln−Ligand system and a Ln−Chromophore system.

### Ln (I)–Ln (II) Bimetallic System in LnMOFs

The traditional method of physically mixing two types of LnMOFs doped with different lanthanide ions, as illustrated in (i) of Figure 1B, is the most straightforward way to obtain dual emission (Chen et al., 2019). Due to the similar chemical properties of lanthanide ions, LnMOF structures can also allow for the incorporation of different types and ratios of lanthanide ions into the same host materials and thus generate characteristic emissions of each lanthanide ion at the same time ((ii) of Figure 1B) (Xia et al., 2017; Su et al., 2018; Ji et al., 2018; Gao et al., 2018; Li et al., 2019a; Othong et al., 2020; Zeng et al., 2020a). Once incorporated into the MOF structure, lanthanide ions can generate emission covering the spectrum region from ultraviolet to near-infrared. Among which, Eu^3+^ and Tb^3+^ ions exhibit strong red and green luminescence, respectively, that can be easily perceived by the naked eye. Therefore, Eu^3+^/Tb^3+^ based MOFs have been widely employed for the construction of dual emission ratiometric fluorescent sensor. For example, chemical fine-tuning the Tb/Eu ratio of raw materials in the synthetic process of Tb/Eu (TATB) results in tunable dual emission from Tb^3+^ and Eu^3+^ ions (Zeng et al., 2020a). Besides serving as the metal nodes, multiple types of lanthanide ions can also be encapsulated into the pore of MOFs as emissive guest species [(iii) of Figure 1B] (Li et al., 2019c; Qiao et al., 2019). For instance, Tb/Eu@bio-MOF-1 material was fabricated via ion exchange between Tb^3+^ and Eu^3+^ cations into the pores of the anionic framework, also resulting in dual emission from Tb^3+^ and Eu^3+^ ions (Zhang et al., 2016b). Noted that the porous microstructure around Tb^3+^ and Eu^3+^ ions also facilitate the absorption and the transportation of analyte and therefore contribute to the effective sensing.

### Ln–Ligand System in LnMOFs

Luminescent behavior of LnMOFs is highly dependent on the efficiency of the antenna effect. The perfectly matched energy levels of ligand and metal result in emission from lanthanide ions dominantly, while poor energy matching leads to almost no lanthanide emission. In the case of partial energy transfer from the ligand to lanthanide ions, both characteristic emissions from lanthanide ion and ligand can be recorded. Such dual emissive Ln–Ligand LnMOF system can be helpful in constructing ratiometric sensors once exposed to analytes (Yang et al., 2017; Yue et al., 2018; Li et al., 2019b; Yin et al., 2019). For example, Qian et al. prepared ZJU-136-Ce_1-x_Eu_x_ with 1,1′; 4′,1″-terphenyl-2′,4,4″,5′-tetracarboxylic acid (TPTC) and Ce^4+^ and Eu^3+^ ions (Yue et al., 2018). ZJU-136-Ce_1-x_Eu_x_ exhibits dual emission from TPTC ligand at 390 nm and Eu^3+^ ions at 617 nm under 320 nm excitation. The introduced Ce^4+^ ions were intended to regulate the energy transfer from TPTC to Eu^3+^ ions. Due to the specific redox reaction between ascorbic acid (AA) and Ce^4+^, the emission intensity of TPTC increased significantly with the simultaneous quenching of Eu ions. Therefore, ZJU-136-Ce_1−*x*_Eu_*x*_ was proved to be useful in ratiometric fluorescence sensing for AA determination.

### Ln–Chromophore System in LnMOFs

MOFs possess pores and channels with highly tunable sizes and shapes. Apart from the intrinsic lanthanide luminescent from the frameworks and the encapsulated Ln^3+^ ions, there are plenty of chromophores such as metal-complex, organic dyes, QDs that can be encapsulated into MOF as chromophore guest (Cui et al., 2015; Gao et al., 2017; Hao et al., 2017; Qin and Yan, 2018; Wang et al., 2019; Yu et al., 2019). For instance, Yu and coworkers developed a water-stable RhB@Tb-dcpcpt host-guest composite by trapping the cationic rhodamine B (RhB) into the anionic framework of [Me_2_NH_2_][Tb_3_ (dcpcpt)_3_(HCOO)]·DMF·15H_2_O via an ion exchange process (Yu et al., 2019). The composite exhibit dual emission from RhB and Tb ions, and therefore allows the realization of sensitive and selective detection toward ciprofloxacin and norfloxacin antibiotics via a luminescent color-changing process. Wang et al. reported a dual-emitting carbon dots@Eu-MOFs for the ratiometric fluorescent detection of Cr(VI) (Wang et al., 2019). Upon reacting with Cr(VI), the fluorescence of carbon dots was quenched while the emission intensity of Eu-MOFs remains unchanged. Therefore, the prepared composites can be employed as self-calibrated probes for Cr(VI).

### Ratiometric Fluorescent Sensing Mechanisms

In a dual-emitting ratiometric fluorescent sensing system, the sensor-analyte interaction can be categorized into the following: analyte modulates by either suppressing (i) or enhancing (ii) the energy transfer from the ligand to lanthanide ions, resulting in reduced or enhanced emission from lanthanide ions. (iii) Analyte modulates the coordination interaction of lanthanide ions. (iv) Analyte modulates the Ln–Ln energy transfer. In general, dual-emitting properties of LnMOF-based sensors show a quick response once in reaction with analyte through the above-mentioned interactions. And the intensity ratio of dual emission can be calculated and then correlated to analytes. In particular scenarios, changes in output emission color can be observed, allowing for fast visual screening of analytes.

**FIGURE 2:**
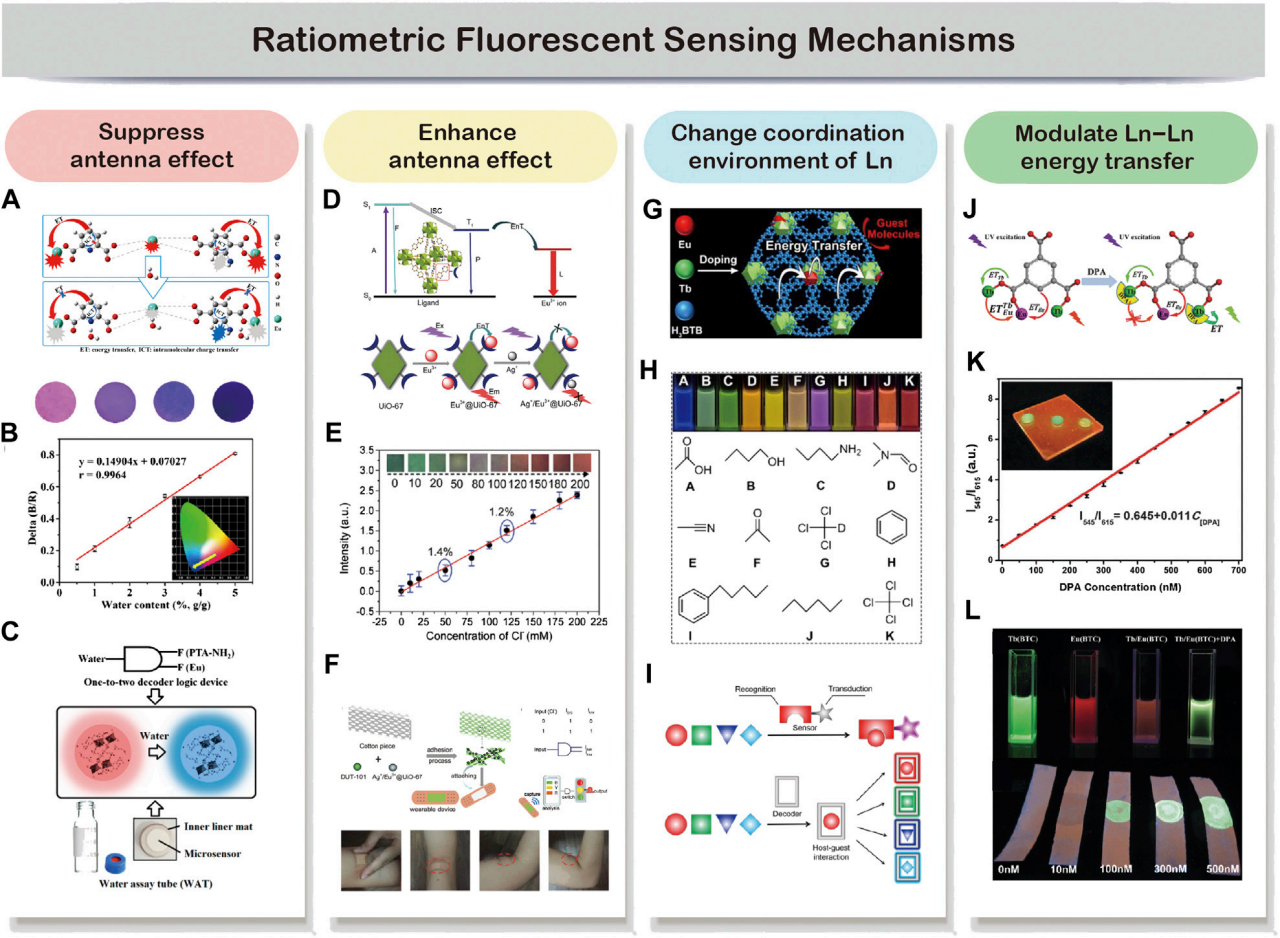
In a dual-emitting system, analyte can be probed through the four commonly employed mechanisms. Analyte interacts with the sensor by suppressing **(A–C)** or enhancing **(D–F)** the antenna effect. Reproduced from Yu et al. (2020) with permission from American Chemical Society and Zhang et al. (2018) with permission from The Royal Society of Chemistry. The analyte may also change the coordination environment of Ln **(G,H)** or modulate the lanthanide-lanthanide energy transfer **(I,J)**. Reproduced from Zeng et al. (2020a) with permission from American Chemical Society and Zhang et al. (2016a) with permission from The Royal Society of Chemistry.

The modulation of antenna effect to quench the lanthanide luminescence is probably the most commonly employed sensing approach by LnMOFs (Cui et al., 2019; Moscoso et al., 2020; Yu et al., 2020). For example, Yu et al. (2020) developed a trace water sensor based on Eu-MOF with the mixed ligand of dipicolinic acid (DPA) and 2-aminophthalic acid (PTA-NH_2_). DPA functioned as the sensitizer for Eu^3+^ ions, while PTA-NH_2_ provides a second emission besides Eu^3+^. The exposure of DPA to water leads to the occurrences of efficient intramolecular charge transfer (ICT) in DPA, which weakens the antenna effect from DPA to Eu^3+^ (Figure 2A). And they observed a “turn-on” blue emission of PTA-NH_2_ with the water content in the MOF and a “turn-off” red emission from Eu^3+^ under 254 nm UV light excitation (Figure 2B). The increase of PTA-NH_2_ blue emission was ascribed to the water-induced ICT fluorescence of PTA-NH_2_. This ratiometric fluorescent sensor exhibits a linear and sensitive response to water in the range of 0–100% (v/v). Therefore, a one-to-two decoder logic device for water assay can be designed (Figure 2C). Based on this dual-emitting ratiometric sensing technique, a paper-based water microsensor was fabricated for portable, longer-term stable, and rapid sensing. In 2017, Yang et al. developed a boric acid functional Eu-MOF probe for the recognition of fluoride ion, which is also based on the suppressed antenna effect (Yang et al., 2017). The insufficient ICT from ligand to Eu^3+^ results in the dual emission from ligand at 366 nm and Eu^3+^ ions at 625 nm under 275 nm excitation. With the presence of fluoride ions, the binding of fluoride to the boron center disrupted the p_π_−*π* conjugation of 5-bop and decreased the intersystem crossing efficiency, and therefore enhance the ligand emission while decreasing the Eu^3+^ emission with increased fluoride concentration. An excellent linear relationship can be observed between the intensity ratio of the dual emission and fluoride concentration in the range from 4 to 80 μM. Moreover, the boric acid exhibits a strong affinity to fluoride ions, while its interaction with other anions is relatively weak, making it perfect for fluoride selective sensing.

Reports on enhancing antenna effect resulted in “turn-on” sensing are rather few comparing to the “turn-off” mechanism discussed above (Karmakar et al., 2019; Fueyo-González et al., 2020; Min et al., 2020; Wang et al., 2020; Yue et al., 2020). However, it is obvious that “turn-on” sensors have advantages over the “turn-off” sensors, especially in the quantitative determination of an analyte in low concentration (Luo et al., 2020). For the “turn-off” sensor, the signal decrease against an already bright background makes it hard for sensitive and accurate sensing. By contrast, “turn-on” luminescent behavior can significantly enhance a weak emission or even create a new emission, reducing the likelihood of false-positive signals while providing much higher sensitivity. Yang et al. developed a “turn-on” ratiometric fluorescent sensor for H_2_S by encapsulating Tb^3+^ ions within the pores of MOF [Cu(HCPOC)_2_]n to form Tb^3+^@Cu-MOF (Zheng et al., 2017). The composite exhibits weak Tb^3+^ emission and strong H_2_CPOC ligand emission because the unsaturated electronic state of Cu^2+^ tends to have a negative effect on the energy transfer from ligand to Tb^3+^ ions. Serving as a strong electron donor, H_2_S can decrease the binding energies of the metal center through its strong affinity for Cu^2+^ ions. Therefore, the luminescence of Tb^3+^ recovered as the result of the restored antenna effect between ligand and Tb^3+^. Meanwhile, the ligand showed a slight decrease in emission with the increase of H_2_S concentration. Therefore, Tb^3+^@Cu-MOF sensor showed excellent sensitivity and selectivity toward H_2_S with a linear response range from 13.25 to 1.6 mM. Another example of enhancing antenna effect-based ratiometric sensing was proposed by Xu and Yan, 2018. They developed a sweat Cl^−^ sensor based on Ag^+^/Eu^3+^@UiO-67 platform. As shown in Figure 2D, Ag^+^ was deliberately introduced into the system via a Ag–N coordination bond to reduce the efficiency of the energy transfer from UiO-67 to Eu^3+^. Upon reaction with Cl^−^, the strong electronic affinity between Ag^+^ and Cl^−^ leads to a “turn-on” luminescence of Eu^3+^. By employing DUT-101 as a reference signal, an obvious output color shift from dark green to bright red can be observed, and a 0.1 mM Cl^−^ limit of detection was claimed (Figure 2E). They further load the two LnMOF-based sensors to a cotton piece and attach it to a band-aid for the demonstration of a wearable Cl^−^ monitoring device (Figure 2F).

Molecules with different coordination capability could cause changes in the coordination environment of lanthanide ions in MOF structures and further affect their emission (Cui et al., 2014; Zeng et al., 2020b). Based on this phenomenon, Zeng et al. developed fast and facile decoding of a broad range of molecules, including homologues, isomers, enantiomers, and deuterated isotopomers by employing Tb/Eu(BTB) as the probe (Zeng et al., 2020b). 1,3,5-benzenetrisbenzoic acid (H_3_BTB) was chosen as the ligand, together with mixed Tb^3+^ and Eu^3+^, to construct Tb/Eu(BTB) probe by a simple one-pot synthesis procedure (Figure 2G). Under 302 nm UV excitation, the probe exhibits red (Eu^3+^), green (Tb^3+^), and blue emissions (BTB) in a single host. Therefore this probe should be ideal for molecular decoding as it involves wide coverage in the visible spectral range. They found out that the ultrahigh decoding capability was realized by fine-tuning the energy transfer pathway in multiple ways, including changing the coordination environment of lanthanide ions within Tb/Eu(BTB) probe. For example, molecular with high coordination capability such as acetic acid could destroy the crystalline structure of LnMOFs and further eliminate the antenna effect. The binding of target molecules to Eu^3+^/Tb^3+^ could change the coordination symmetry of the LnMOFs and modulate the energy of singlet or triplet excited states of BTB, affecting the energy transfer from BTB to Eu^3+^/Tb^3+^. As shown in Figure 2H, this ratiometric molecular decoding strategy enables a full-color readout and visual decoding capability. Conventional molecular decoding strategy requires a tailor-designed probe for a specific sensing target, which is time-consuming and labor-intensive. The reported probes can accommodate a diverse range of molecules, while each gives a different output signal as the result of specific host-guest interaction (Figure 2I). This decoding strategy allows for multiple-target differentiation by using only a single probe, significantly promoting its widespread usability.

Modulating the Ln–Ln energy transfer is a straightforward way to tune the emission ratio of two lanthanide ions and correlate with the concentration of analytes (Zhang et al., 2016a; Zhao et al., 2019; Wu et al., 2020b; Feng et al., 2020). For example, Zhang and coworkers developed a dual-emitting Tb/Eu(BTC) probe for the rapid detection of dipicolinic acid (DPA) biomarker (Zhang et al., 2016a). Since DPA can chelate with Tb^3+^, the energy transfer from DPA to Tb^3+^ becomes dominant while the Tb-to-Eu energy transfer is interrupted (Figure 2J). The concentration of DPA can be linearly correlated well with the ratio *I*_545_/*I*_613_ in the 50–700 nM concentration range with the estimated detection limit of 4.55 nM (Figure 2K). And the color output of Tb/Eu(BTC) sensor changes from orange-red to yellow-green after adding DPA in the solution or dropping onto test strips (Figure 2L). Another great example of using an analyte to modulate Ln–Ln energy transfer is for ratiometric temperature sensing (Yang et al., 2020c; Feng et al., 2020). Feng et al. developed Eu_0.0025_Tb_0.9975_-BABDC-PBMA membrane. H_2_BABDC is an ideal “antenna chromophore” to sensitize both Eu^3+^ and Tb^3+^, and therefore generate dual emission (Feng et al., 2020). They found that the intensity ratio of ^5^D_4_→^7^F_5_(Tb^3+^)/^5^D_0_→^7^F_2_(Eu^3+^) can be linear related to the temperature from 90 to 240 K. And the energy transfer from Tb^3+^ to Eu^3+^ was responsible for the unique temperature-dependent luminescent behavior. Benefiting from the tunable architectures of this probe, they then developed a free-standing and homogeneous membranous ratiometric luminescent thermometer with excellent mechanical properties. And the thermometer was tested to be stable even under harsh conditions, pushing forward the real application of LnMOF-based thermometers.

## Conclusions and Perspective

This mini review provides a brief overview of the strategies to construct a dual-emitting LnMOF-based ratiometric fluorescent sensor. By virtue of the structural versatility, luminescent turnability, unique sensor-analyte interaction, and the possible fabrication into smart-devices make LnMOF an excellent candidate for efficient ratiometric fluorescent sensing. This technique relies on the change in the emission intensity of two emission bands, allowing for precise, quantitative, and real-time analysis. And the direct witness of the color change by the naked eye could be a more facile way for the determination of analytes. Despite the undeniable merits of LnMOF-based ratiometric fluorescent sensors, there are still many challenges to overcome. The stability, especially the water stability of the LnMOF-based sensors, needs to be further enhanced to avoid the structural breakdown before completing the sensing in the aqueous environment or other sensing media. Most of the LnMOFs sensing experiments were still conducted in solution. The development of LnMOF films or membranes as portable devices while maintaining the original stability and ratiometric sensing capability can be meaningful during the exploration of their practical applications. The design and fabrication of LnMOFs in nanometer scale is still in their infancy. Further effort can be devoted to preparing nanostructured LnMOFs, which holds great promise in bioimaging, diagnosis, and therapy. Most of the LnMOF-based sensors are still based on the quenching mechanism. Research endeavors are preferred to devote toward the rational design of “turn-on” especially “turn-on” ratiometric fluorescent sensors.

## Author Contributions

All authors listed have made a substantial, direct, and intellectual contribution to the work and approved it for publication.

## Funding

This work was supported by the National Natural Science Foundation of China (Nos. 51802198 and 51902355), the start-up Grant of Sun Yat-sen University.

## Conflict of Interest

The authors declare that the research was conducted in the absence of any commercial or financial relationships that could be construed as a potential conflict of interest.
